# Genome sequences published outside of *Standards in Genomic Sciences,* December 2011

**DOI:** 10.1601/sigs.2495686

**Published:** 2011-12-30

**Affiliations:** 1 Editorial Office, Standards in Genomic Sciences and Department of Microbiology, Michigan State University, East Lansing, MI, USA

Oranmiyan W. Nelson^1^ and George M. Garrity^1^

The purpose of this table is to provide the community with a citable record of publications of ongoing genome sequencing projects that have led to a publication in the scientific literature. While our goal is to make the list complete, there is no guarantee that we may have omitted one or more publications appearing in this time frame. Readers and authors who wish to have publications added to subsequent versions of this list are invited to provide the bibliographic data for such references to the SIGS editorial office.

**Phylum *Euryarchaeota***
*Halobiforma lacisalsi* AJ5, sequence accession AGFZ01000000 [1]
*Thermococcus sp.* strain AM4, sequence accession CP002952 [2]
**Phylum *Proteobacteria***
*Acidithiobacillus thiooxidans* ATCC 19377, sequence accession AFOH01000000 [3]
*Acinetobacter sp.* strain D499, sequence accession AGFH01000000 [4]
*Azospirillum brasilense* Sp245, sequence accession HE577327 (chromosome), HE577328 (p1), HE577329 (p2), HE577330 (p3), HE577331 (p4), HE577332 (p5), HE577333 (p6) [5]
*Azospirillum lipoferum* strain 4B, sequence accession FQ311868 (chromosome), FQ311869 (p1), FQ311870 (p2), FQ311871 (p3), FQ311872 (p4), FQ311873 (p5), FQ311874 (p6) [5]
*Escherichia coli* F18, sequence accession AGTD01000000 [6]
*Escherichia coli* K88, sequence accession CP002729 (chromosome), CP002730 (pUMNK88_K88), CP002731 (pUMNK88_IncI1), CP002732 (pUMNK88_Ent), CP002733 (pUMNK88_Hly), and HQ023862 (pUMNK88) [6]
*Escherichia coli* Strain CE10, sequence accession CP003034 to CP003038 [7]
*Gluconacetobacter xylinus*, NBRC 3288, sequence accession AP012159 through AP012166 [8]
*Halomonas sp.* Strain HAL1, sequence accession AGIB00000000 [9]
*Methylomonas methanica* MC09, sequence accession CP002738 [10]
*Novosphingobium nitrogenifigens* Y88, sequence accession [11]
*Pelagibacterium halotolerans* B2, sequence accession CP003075 (chromosome), CP003076 (plasmid) [12]
*Pseudomonas aeruginosa* PAO1, sequence accession GSE34141 [13]
*Pseudomonas putida* Idaho, sequence accession AGFJ01000000 [14]
*Sphingomonas elodea* ATCC 31461, sequence accession AGFU01000000 [15]
**Phylum *Firmicutes***
*Acidaminococcus intestini* RYC-MR95, sequence accession CP003058 [16]
*Lactobacillus rhamnosus* strain CASL, sequence accession AFYD00000000 [17]
*Pseudomonas aeruginosa* NCGM2.S1, sequence accession AP012280 [18]
**Phylum *Actinobacteria***
*Candidatus* Frankia datiscae, sequence accession NC_015656 [19]
*Corynebacterium pseudotuberculosis* Strain CIP 52.97, sequence accession CP003061 [20]
*Propionibacterium acnes* Type II Strain ATCC 11828, sequence accession CP003084 [21]
*Streptomyces chartreusis* NRRL 12338, sequence accession AGDE00000000 [22]
*Streptomyces chartreusis* NRRL 3882, sequence accession AGDD00000000 [22]
*Streptomyces lysosuperificus* ATCC 31396, accession number AGDC00000000 [22]
*Streptomyces sp.* Strain Wigar10, sequence accession AGDF01000000 [23]
**Phylum *Spirochaetes***
*Borrelia afzelii* PKo, sequence accession CP002933 (Chromosome), CP002942 (Ip17), CP002943 (Ip28-2), CP002944 (Ip28-3), CP002945 (Ip28-4), CP002946 (Ip28-7), CP002947 (IP28-8), CP002949 (Ip38), CP002950 (Ip54), CP002934 (cp26), CP002937 (cp32-1), CP002938 (cp32-3), CP002939 (cp32-5), CP002940 (cp32-7), CP002940 (cp32-9), CP002948 (cp32-10), CP002935 (cp32-11), CP002936 (cp32-12) [24]
*Borrelia afzelii* ACA-1, sequence accession ABCU02000001-2 (Chromosome), CP001239 (Ip17), CP001238 (Ip28-1), CP001244 (Ip28-2), CP001241 (Ip28-3), CP001249 (Ip28-4), CP001242 (Ip-7), CP001246 (Ip38), CP001247 (Ip54), CP001250 (cp26), CP001243 (cp32-1), CP001237 (cp32-3), CP001240 (cp32-4), CP001248 (cp32-5), CP001245 (cp32-10) [24]
*Borrelia garinii* PBr, sequence accession ABJV02000001-5 (Chromosome), CP001309 (Ip17), CP001301 (Ip25), CP001310 (Ip28-1), CP001307 (Ip28-3), CP001304 (Ip28-4), CP001311 (Ip28-7), CP001302 (Ip36), CP001308 (Ip54), CP001305 (Ip26), CP001303 (Ip32-5), CP001306 (Ip32-10) [24]
*Borrelia garinii* Far 04, sequence accession ABPZ02000001-33 (Chromosome), CP001315 (Ip17), CP001317 (Ip25), CP001316 (Ip28-1), CP001314 (Ip36), CP001318 (Ip54), CP001319 (Ip26), CP001320 (Ip32-10) [24]
Non-Bacterial genomes
B1 Human Adenovirus HAdV-16 strain E26, sequence accession JN860680 [25]
B1 Human Adenovirus HAdV-3/16, sequence accession JN860678 [25]
B1 Human Adenovirus HAdV-3+7, sequence accession JN860679 [25]
B1 Human Adenovirus HAdV-7d2, sequence accession JN860677 [25]
B1 Human Adenovirus HAdV-7h, sequence accession JN860676 [25]
*Bacillus cereus* bacteriophage BCP78, sequence accession JN797797 [26]
Circoviridae member (not yet validated), sequence accession JF803741 [27]
Coccolithovirus Emiliania huxleyi Virus 203, sequence accession JF974291 [28]
*Cryptococcus gattii* BC, sequence accession SRP006436 [29]
Erwinia amylovora plasmid pEI70, sequence accession CP002951 [30]
*Mortierella alpina*, sequence accession ADAG00000000 [31]
Parvovirus Aj-BtPV-1, sequence accession JN860679 [32]
Parvovirus Eh-BtPV-1, sequence accession JN860679 [32]
Penicillium marneffei PM1, sequence accession AGCC00000000 [33]
Pseudomonas fluorescens phage OBP, sequence accession JN627160 [34]
Salmonella bacteriophage SPN3US, sequence accession JN641803 [35]
Tailam virus, sequence accession JN689227 [36]
